# Group Imagery Rescripting on Childhood Memories Delivered *via* Telehealth: A Preliminary Study

**DOI:** 10.3389/fpsyt.2022.862289

**Published:** 2022-06-23

**Authors:** Katia Tenore, Alessandra Mancini, Olga Ines Luppino, Francesco Mancini

**Affiliations:** ^1^Associazione Scuola di Psicoterapia Cognitiva (APC-SPC), Rome, Italy; ^2^Department of Human Sciences, Marconi University, Rome, Italy

**Keywords:** imagery rescripting, maladaptive beliefs, autobiographical memories, memory realism, emotional needs, affective state, needs’ satisfaction

## Abstract

**Background:**

Imagery Rescripting (ImR) has proved to be effective in the treatment of different mental disorders as an integral part of broader clinical protocols or as a standalone technique. ImR has also been successfully incorporated as part of group Schema Therapy treatment; however, to the best of our knowledge, it has never been assessed as a standalone technique in a group setting.

**Aim:**

In this study, we focused on ImR delivered *via* telehealth in groups and we aimed to assess whether group ImR is effective in responding to basic emotional needs, in changing participants’ affective state, and in reducing dysfunctional beliefs. We also wanted to assess whether memory realism is associated with a greater effectiveness of the technique.

**Methods:**

A total of 52 participants were presented with 3 ImR sessions on childhood memories related to the current dysfunctional belief that elicited more suffering.

**Results:**

The technique was effective in facilitating the retrieval of a memory in almost the entire sample (in the range of 92.3–100%). Overall, memory realism values (level of vividness, ability to immerse, and participants’ distance from the images) were high in all three sessions. Almost all participants were reported having their needs met during ImR (89.7%). Importantly, need satisfaction was associated with the ability to immerse in the image. In addition, the intensity of the dysfunctional belief decreased significantly from pre-test to Session 3. The technique also changed the affective state, reducing arousal. Importantly, we also observed a general reduction in shame levels from the first to the third session.

**Conclusion:**

A telehealth delivered ImR group intervention on childhood memories provides cognitive and emotional improvement. Along with the ability to satisfy the patient’s basic emotional needs, the technique seems to be effective in modifying maladaptive beliefs encapsulated in memory.

## Introduction

Imaginative techniques have been used in therapeutic contexts, for several centuries and have now been extensively integrated into the Schema Therapy (ST) approach (1, 2). Imagery Rescripting (ImR) is a therapeutic technique that aims to reduce the distress associated with negative memories of early aversive experiences. It consists of prompting patients to rescript the autobiographical memory in order to satisfy their unmet needs. Although these basic needs are considered to be important in the genesis of maladaptive beliefs in Schema Therapy, studies specifically monitoring in which specific emotional needs are frustrated are scarce.

One of the salient features of ImR is its effectiveness in the treatment of different mental disorders. Over the past 10 years, abundant evidence proving efficacy has been collected, using ImR as both an integral part of broader clinical protocols and as a standalone technique (3, 4).

When considered as part of broader clinical protocols, ImR has proved to be effective in reducing the symptoms of posttraumatic stress disorder (PTSD) (5–7), social phobia (8, 9), and personality disorders (10). However, in these studies, ImR has not been experimentally isolated from cognitive restructuring.

The technique has proved to be effective also as a standalone technique in the treatment of PTSD (11–15), social phobia (16, 17), body dysmorphic disorder (18, 19), bulimia (20), depression (21, 22), and obsessive-compulsive disorder (OCD) (23–26). Recently, ImR has also been found to be effective at treating trauma-affected voice hearers (27).

The aforementioned studies delivered ImR individually, but ImR has also been successfully incorporated into Group Schema Therapy treatment of borderline personality disorder and eating disorder (28–32). The application of Group Schema Therapy has been described in the work by Farrel et al. (32).

However, to the best of our knowledge, the efficacy of group ImR on early childhood memories as a standalone technique has not yet been studied. Thus, in this study, we focused on ImR delivered in groups.

Initially, this research was conceived with the idea of delivering group ImR in person, but the spread of the COVID-19 pandemic forced the authors to perform the procedure *via* telehealth. A recent study supports the delivery of ImR *via* telehealth *s*howing that, as long as privacy is granted, it is not less effective than face-to-face delivery (33). Group telehealth imagery setting might have the clear advantage of containing therapy costs. Moreover, it enables geographical distances to be reduced and thus allows access to population groups from more rural areas, which are often prevented from accessing clinical and research centers.

Notably, a growing body of literature has shown that the ability to become immersed in the autobiographical memory affects ImR outcome (34–37). Therefore, in this study, we assessed whether memory realism (level of vividness, ability to immerse, and participants’ distance from the images) was associated with a greater effectiveness of the group technique in meeting the subject’s core needs and in changing the dysfunctional belief attached to the memory.

Thus, the main purposes of this research were to verify the effectiveness of a group ImR intervention *via* telehealth. Specifically, we aimed to assess whether group ImR is effective in (i) responding to the basic emotional needs of participants as defined by ST, (ii) reducing dysfunctional beliefs and, and (iii) changing participants’ affective state.

In line with previous observations, we hypothesize that group ImR is effective in producing a cognitive and emotional improvement. In addition, we expect memory realism features to affect ImR effectiveness in meeting participants’ needs.

## Materials and Methods

### Participants

The sample was composed of 52 participants (48 F). Participants were recruited among the students and alumni of the School of Cognitive Psychotherapy (SPC) of Rome (Italy). Specifically, the study was advertised through the School newsletter. Those who were interested in taking part in the study could volunteer by contacting the authors. Importantly, participants were only included if they did not have previous experience with ImR (e.g., during therapy) or if they have not been theoretically introduced to this technique (e.g., during classes). Along with age, sex, and education level, participants were asked if they have ever been diagnosed with a psychiatric disorder, if they were currently (or had in the past) undertaking psychotherapy, and if they were taking psychiatric medication. Participants were from 10 different Italian regions, and mean age was 31.5 (*SD* = 5.06, range = 26–60). Notably, 51% had a university degree, 47.1% were specialized or held a PhD, and 2% had a high school diploma. Moreover, 13.46% of the entire sample was reported to have received a psychiatric diagnosis (i.e., three panic disorder; one anorexia nervosa; one cyclothymia; two major depressive disorder). Notably, 22% of the sample was currently undertaking psychotherapy and 2% was taking psychiatric medication.

### Ethics

All participants were given a digital informed consent form and gave their informed consent prior to inclusion in the study by choosing to proceed with the surveys. Procedures were carried out in accordance with the principles of the Declaration of Helsinki and were approved by the Guglielmo Marconi University Ethical Committee (Protocol Date: 24 March 2020).

### Procedure

All participants responded to the questionnaire online through the survey platform Question Pro.^1^ They were recruited by means of an advertisement, circulated by SPC. Participants did not receive any form of payment for their participation in the study. After signing the informed consent, they completed the Young Schema Questionnaire- Short Form (YSQ-SF) (38) and the Personality Belief Questionnaire- Short Form (PBQ-SF) (39). In addition, they were asked to choose the statement that evoked in them the greatest suffering among the 140 YSQ-SF and PBQ-SF items. Participants were then presented with 3 ImR sessions, as in previous studies (16, 26), starting from memories related to these statements (i.e., the dysfunctional belief) which currently elicits more suffering.

A week after completing the questionnaires, participants received the first ImR session. The three ImR sessions were delivered weekly.

Different ways of conducting the study of ImR have been proposed, but, in this study, we employed the three-stage one proposed by Arntz and Weertman (40), adapting it to the group setting which is in line with the work by Farrell et al. (32).

Participants were asked to close their eyes and imagine themselves in a previously identified safe place. Then, they were asked to think about the last time they experienced the negative self-belief they selected among the 140 YSQ and PBQ items and to focus on the emotional and somatic experience connected to this belief. This method of emotional activation is designed to bring about a connection to the memory of a childhood event. It is called a “bridge” (41), because it enables the emotions and/or somatic experience of a present event to connect with a memory of a past event in which the same feelings were experienced.

The memory retrieved was then revisited by the participant in detail, from the perspective of the participant as a child. After reliving the scene from the child’s perspective, the therapist asked the participant to change the point of view and to relive the past experience again, this time observing it through the eyes of the participant’s adult-self. In this study, the participant enters the image as an adult and performs all the actions that lead to the resolution of suffering and to the satisfaction of the child-self’s frustrated needs (e.g., protecting, taking care, being empathic, and setting limits).

Importantly, to increase the replicability, in each session, participants heard the same script (read by the first author of this study), which was adapted from the work of Farrell et al. (28). The script consisted of the following steps: (1) Instructions; (2) group safe place; (3) focusing on the recent situation that activates the belief; (4) floating back; (5) rescripting the memory emerged *via* the bridge emotion; and (6) back to the safe place (see Supplementary Appendix).

Subsequently, they were presented with an *ad hoc* questionnaire investigating memory features (see below). Before and after each ImR session, they assessed their affective state on the positive affect and negative affect scale (PANAS) (42).

### Measures

#### The Young Schema Questionnaire Short Form

The Young Schema Questionnaire short form (YSQ-SF) (38) is a 75-item self-report inventory that assesses 15 Early Maladaptive Schemas (EMS) proposed by Young et al. (1). Each item in the questionnaire is a statement based on a maladaptive belief as defined by schema theory. Respondents are asked to rate the degree to which they agree with the statements on a 6-point Likert scale (1–6). A mean score is calculated for each EMS, a higher score representing a higher endorsement of the EMS in question. Since no Italian validation of this reduced version of the YSQ is available, we selected 75 items on the basis of the English validation study (43), which reported the Cronbach’s alpha coefficient ranging from 0.80 to 0.93.

#### The Personality Belief Questionnaire Short Form

The Personality Belief Questionnaire Short form [PBQ-SF; (39)] is a 65-item self-report inventory developed as a clinical and research instrument to assess dysfunctional beliefs associated with personality disorders, as described by the Diagnostic and Statistical Manual for Mental Disorders (44). The measure was developed starting from the original PBQ 126-items’ form (45). A total of 65 items were selected among the corresponding items contained in the 126 items’ PBQ Italian validation (46). The inventory assesses the specific beliefs related to 9 personality disorders, including Avoidant, Dependent, Passive-aggressive, Obsessive-compulsive, Antisocial, Narcissism, Histrionic, Schizoid, and Paranoid. PBQ-SF is a self-report Likert-type questionnaire that is scored from 0 (“I don’t believe it at all.”) to 4 (“I believe it totally.”). The Cronbach’s alpha coefficient for the total PBQ-SF score was α = 0.97 (39). The Cronbach’s alpha values for the single scales were as follows: Avoidant (0.84), Dependent (0.89), Passive-Aggressive (0.86), Obsessive-Compulsive (0.90), Antisocial (0.80), Narcissistic (0.83), Histrionic (0.89), Schizoid (0.79), and Paranoid (0.91).

#### The Positive and Negative Affect Schedule

The PANAS consists of 10 negative and 10 positive mood terms and a 5-point Likert-type response scale from 1, very slightly or not at all, to 5, extremely, with scale scores ranging between 10 and 50 (42). In this study, the scale scores were calculated to range between 0 and 40 (i.e., scale score −10), and respondents were asked to indicate how often they felt that way in general. Evidence of the validity of the Italian version is obtained from the pattern of the relationship with personality and depression measures. As reported by Terracciano (47), the relationship between PA and Extraversion and NA and Neuroticism found with this Italian sample replicated the American findings and the Cronbach’s alpha coefficient score ranged from 0.83 to 0.87 (48). The positive affect subscale reflects the extent to which a person feels interested, excited, strong, enthusiastic, proud, alert, inspired, determined, attentive, and active. On the other hand, the negative affect subscale includes descriptors such as stressed, upset, guilty, scared, hostile, irritable, ashamed, nervous, jittery, and afraid.

#### Memory Features Questionnaire

Memory features were investigated by means of an *ad hoc* questionnaire. Specifically, participants were asked if the technique was able to facilitate the retrieval of a memory (binary yes/no response). Furthermore, they were asked to report on a five-point scale how vivid the memory retrieved was, how much they were able to emotionally immerse into the memory, and how much far the images were. Subsequently, they were asked to report their age at the time of the memory and indicate which needs were frustrated in this memory, among the five core emotional needs described by Young et al. (1). Specifically, in the questionnaire, core emotional needs were described as follows (*translated from the Italian*): (i) “Secure attachment (protection, safety, stability, care, and acceptance)”; (ii) “Autonomy, Sense of competence, and Sense of identity”; (iii) “Realistic limits”; (iv) “Freedom to express emotions”; and (v) “Spontaneity and play.” Participants could indicate whether the need was frustrated providing a yes/no answer. After each ImR session, participants further assessed on a three-point scale to what extent they felt their needs were met during the rescripting (“0” = not at all; “1” = in part; “2”completely). Finally, before Session 1 and after Session 3, participants were assessed on a six-point scale to determine how much they believed in the dysfunctional belief they identified in the pre-test phase.

### Data Analysis

Descriptive statistics were employed to determine the percentage of participants who were able to retrieve a childhood memory; participants’ mean age (and standard deviations) in the memories and memory realism measures (mean and standard deviations across session). In addition, we assessed with which frequency participants reported unmet needs in each of the five domains and the percentage of participants who reported that their needs were met through rescripting. Furthermore, with the aim to test the association between realism measures and needs’ satisfaction, we performed Spearman correlations between average realism measures and average needs’ satisfaction scores.

Putative differences in the strength of the dysfunctional belief between the pre-test and the third ImR session were assessed by means of a paired sample *t-*test. Finally, differences in participants’ affective state (i.e., PANAS values) before and after each session were assessed by means of a series of paired sample *t*-tests.

## Results

### Descriptive Statistics

As shown in Table 1, the technique was effective in facilitating the retrieval of a memory in almost the entire sample in all the three sessions. The earliest memory retrieved was of being 3 years old, the oldest was of being 16 years old. In addition, mean memory realism values were good in all three sessions. No differences in these measures were found across sessions other than participants, which revealed that they were able to immerse themselves in the memory more effectively in session 1 [*F*_(1.98, 98.86)_ = 13, *p* < 0.001, η*_*p*_*^2^ = 0.206, *Post hoc* tests: all Adj.*ps* < 0.001]. When asked about the type of unmet need in the memory, the majority of participants reported unmet needs in the Secure Attachment domain, followed by the Freedom to express emotions and needs’ domain and the Autonomy/sense of identity domain. Fewer unmet needs were reported in the Spontaneity and Play domain and in the Realistic Limit domain. Almost the entire sample was reported to be able to meet their emotional needs through rescripting (89% on average).

**TABLE 1:** Descriptive statistics.

	S1		S2		S3		Average
Descriptive measures	M	SD	M	SD	M	SD	
Age in the memory	7.37	2.35	8.36	2.90	8.27	2.99	8
Vividness (1–5 Likert scale)	3.37	0.81	3.19	0.79	3.24	1	4.3
Ability to immerse	3.5	0.7	3.06	0.75	3.06	0.88	3.2
Distance of the image	2.43	0.83	3.06	0.88	2.65	1.04	2.71
Needs satisfaction (1–3 Likert scale)	2	0.56	1.38	0.56	1.79	0.54	1.72
	%		%		%		Average%
Success to retrieve a memory	100		92.3		96.2		96.2
Overall satisfaction of needs (Y/N)	96.2		86.5		86.5		89.73
Frustrated needs	%		%		%		Average%
Secure attachment	69.2		61.5		63.4		64.7
Autonomy, competence, identity	21.2		27		25		24.4
Realistic limits	3.8		1.9		3.8		3.2
Freedom to express needs	23.1		32.7		38.5		39
Spontaneity and Play	5.8		7.7		13.5		9

*Mean and standard deviation values are reported for the age in the memory, for memory realism measure and for the level of needs’ satisfaction. Percentages of participants who were able to retrieve a memory and to satisfy their emotional needs through rescripting are shown. Finally, the table reports the % of participants reporting to have needs frustrated in each domain.*

### Predictors of Needs’ Satisfaction

Spearman correlation coefficients and *p*-values are reported in Table 2. We found an association between the ability to immerse in the memory and needs’ satisfaction levels (ρ = 0.458, *p* = 0.000). No other significant association was found.

**TABLE 2:** Results of the Spearman correlation between memories average realism measures and needs satisfaction levels.

Correlation between average realism measures and needs satisfaction levels		
	**Rho**	***p***
Vividness	0.206	0.135
**Ability to immerse**	**0.458**	**<0.0001**
Distance	−0.162	0.241

*Significant differences are highlighted in bold.*

### Dysfunctional Belief

The paired-sample *t-*test performed on dysfunctional belief measures determined a significant difference between Session 1 (*M* = 4.24, *SD* = 1.11) and Session 3 (*M* = 3.02, *SD* = 1.22), indicating a significant decrease of the dysfunctional belief after 3 ImR weekly sessions (*t* = 5.20, *p* < 0.001, and Cohens’*d* = 0.74).

### Emotional State Measures (Positive Affect and Negative Affect Scale)

The repeated measures *t*-tests performed on PANAS measures determined some changes in the affective state after each ImR session. After Session 1, participants felt significantly more distressed, strong, inspired, proud, less ashamed, scared, active, attentive, and enthusiastic. After Session, 2 participants felt significantly less attentive, active, and ashamed. After Session 3, participants felt significantly stronger and prouder and less alerted, active, attentive, distressed, irritable, nervous, and ashamed. Overall, Table 3 shows the complete results of the *t-*tests.

**TABLE 3:** Results of the paired sample *t-*test for each affective state measured by the PANAS.

Session 1				Session2			Session 3		
PRE/POST	*t*	*p*	Cohen’s *d*	*t*	*p*	Cohen’s *d*	*t*	*p*	Cohen’s *d*
Jittery	1.399	0.168	0.1941	0.682	0.498	0.0946	1.749	0.086	0.2425
Alerted	0.551	0.584	0.0764	1.825	0.074	0.2531	2.675	**0.010**	0.3710
Distressed	−3.597	**<0.001**	−0.4988	−1.906	0.062	−0.2643	2.108	**0.040**	0.2923
Attentive	3.200	**0.002**	0.4438	4.217	**<0.001**	0.5848	2.751	**0.008**	0.3815
Active	4.947	**<0.001**	0.6860	4.067	**<0.001**	0.5641	2.442	**0.018**	0.3387
Determined	0.000	1.000	0.0000	1.530	0.132	0.2122	0.313	0.755	0.0435
Enthusiastic	2.540	**0.014**	0.3523	1.956	0.056	0.2713	0.903	0.371	0.1252
Excited	1.925	0.060	0.2669	1.477	0.146	0.2048	1.098	0.278	0.1522
Strong	−2.635	**0.011**	−0.3654	−1.376	0.175	−0.1908	−2.615	**0.012**	−0.3627
Afraid	−0.726	0.471	−0.1007	−1.399	0.168	−0.1941	1.071	0.289	0.1485
Interested	1.399	0.168	0.1941	0.535	0.595	0.0742	0.851	0.399	0.1179
Irritable	−0.136	0.892	−0.0189	1.541	0.129	0.2137	3.503	**<0.001**	0.4857
Inspired	−2.232	**0.030**	−0.3096	−0.155	0.878	−0.0215	0.000	1.000	0.0000
Nervous	1.428	0.159	0.1981	1.993	0.052	0.2763	2.535	**0.014**	0.3515
Proud	−3.421	**0.001**	−0.4745	−1.307	0.197	−0.1812	−2.599	**0.012**	−0.3604
Hostile	−1.218	0.229	−0.1689	0.724	0.472	0.1004	1.767	0.083	0.2450
Guilty	−0.423	0.674	−0.0587	0.685	0.497	0.0950	1.935	0.059	0.2683
Upset	−1.137	0.261	−0.1577	−0.629	0.532	−0.0872	−0.574	0.569	−0.0795
Scared	−3.195	**0.002**	−0.4431	−1.531	0.132	−0.2124	0.622	0.537	0.0862
Ashamed	2.329	**0.024**	0.3230	3.120	**0.003**	0.4326	3.056	**0.004**	0.4238

*Significant differences are highlighted in bold.*

To observe putative (increasing or decreasing) trends in PANAS scores between sessions, we compared mean PANAS values after S1, S2, and S3, by means of a repeated measure ANOVA. Only the emotions that resulted in significant changes after the ImR in each session (according to the *t-*test results, see Table 3) were included in the latter analysis.

**FIGURE 1:**
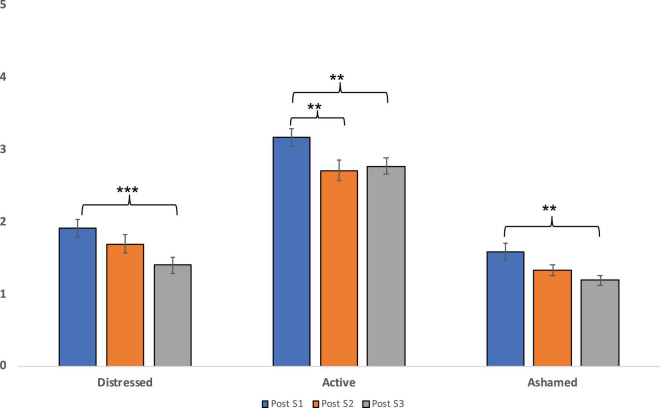
The differences in mean post ImR scores of distress, activation, and shame levels between sessions. **p < 0.01; ***p < 0.001.

The 10 repeated measure ANOVAs, with a Greenhouse-Geisser correction, performed separately on each PANAS emotion which significantly changed after ImR, determined a significant difference between sessions in Distress scores [*F*_(2, 102)_ = 8.36, *p* < 0.001, η^2^*_*p*_* = 0.05]. *Post hoc* tests using the Bonferroni correction revealed that distress decreased from S1 (*M* = 1.92; *SD* = 0.86) to S3 (*M* = 1.40; *SD* = 0.82) (Adj.*p* < 0.001). No significant difference was observed between S1 and S2 (*M* = 1.69; *SD* = 0.91) and between sessions 2 and 3 (all *p*s < 0.05, NS). Moreover, a significant change in Active scores was observed [*F*_(2, 102)_ = 7.07, *p* = 0.001, η^2^*_*p*_* = 0.05]. Bonferroni correction revealed that participants were reported to be more active in S1 (*M* = 3.17; *SD* = 0.87) as compared to S2 (*M* = 2.71; *SD* = 1.02) (Adj.*p* = 0.008) and S3 (*M* = 1.40; *SD* = 0.82) (Adj.*p* = 0.008). No significant difference was observed between sessions 2 and 3 (all *p*s < 0.05, NS). Finally, a significant change was revealed in Shame scores [*F*_(2, 102)_ = 7.21, *p* = 0.001, η^2^*_*p*_* = 0.206]. Specifically, participants were reported to feel less ashamed after S3 (*M* = 1.19; *SD* = 0.52) as compared to post scores in S1 (*M* = 1.58; *SD* = 0.87) (Adj.*p* = 0.005). No significant difference was observed between S1 and S2 (*M* = 1.33; *SD* = 0.55) and between Sessions 2 and 3 (all *p*s < 0.05, NS). No other significant effect was found (all *p*s < 0.05, NS). Significant results are reported in Figure 1.

## Discussion

The aim of this study was to assess the effectiveness of a telehealth delivered ImR group intervention on childhood memories, in producing cognitive and emotional improvement. One of the first questions, we attempted to reply, was if ImR group intervention would be able to make participants retrieve memories. From the data, it is found that group ImR was effective in facilitating the retrieval of childhood memories. Monitoring the types of frustrated needs was not the main aim of this study but it is nevertheless interesting given their central role in the genesis of maladaptive beliefs in Schema Therapy. Specifically, memories were associated mostly with the frustration of secure attachment needs, than with the need for autonomy, competence, and sense of identity, and subsequently with the need to express one’s own needs and emotions, the need for spontaneity and play, and finally with the need for realistic limits. Group ImR was effective in facilitating needs’ satisfaction in almost the totality of our sample. This result is very important because in ImR individual delivery, patients’ need satisfaction is the ultimate goal of the intervention, which is concluded by the therapist only when participants report that they are emotionally satisfied (36). Along with the ability to satisfy the patient’s basic emotional needs, the technique seems to be effective in modifying maladaptive beliefs encapsulated into memory as already reported by Lee and Kwon (16), Wild et al. (8, 9), Cooper (20), in clinical samples, and by Otera et al. (49) in a non-clinical sample. The change in the maladaptive belief is observable particularly between the pre-test and the second and third sessions. This result is consistent with previous studies that show that the efficacy of the ImR interventions is notable a week after the first intervention (4, 50). As several studies have already proposed, three ImR sessions seem to be effective in modifying dysfunctional intakes or symptomatic conditions (16, 26).

Memories evoked through the group script were generally described as vivid and close, and participants stated to have been able to immerse themselves in the image. Importantly, this latter measure was strongly associated with the perception of having one’s own needs satisfied. This result is in line with a recent study by Looney et al. (37), who compared ImR features between high and low responders, in a sample of PTSD participants. The authors found that the new elements incorporated into the imagery are perceived as more vivid in high responders than in low responders. Therefore, considering the growing interest in the delivery of therapeutic techniques outside the classical therapeutic setting (51), helping the client to immerse themselves in memory seems to be important in the memory rehearsal and in satisfying the unmet emotional needs. This is in line with the study showing larger effects when app-based interventions are combined with therapist support (52).

Finally, our data show that the intervention brought about a change in the affective state. In the three sessions, there was a reduction in attention and activation levels. It would seem that ImR sessions reduced arousal bringing participants to a state of greater calm. This effect could be associated with progressively reduced fatigue in processing the emotional content related to target memories. This result is in line with previous studies indicating that ImRs reduce state stress symptoms (53).

Importantly, we also observed a general reduction in shame levels from the first to the third sessions. To the best of our knowledge, studies directly investigating the effect of group ImR on the affective state are very limited. However, previous studies reported a pre- and post-therapy reduction in shame and anxiety levels in participants with eating disorders and mixed personality disorders (29, 30). Our results suggest that increased positive emotionality might constitute a mechanism for the effectiveness of ImR, which is in line with the study by Dibbets et al. (54).

## Conclusion

In conclusion, this study suggests that group ImR is a promising technique. However, it underlines the importance of monitoring the degree of immersion in the memory, which not only facilitates the memory rehearsal, but also makes the memory more vivid and affectively characterized. This provides evidence for which clinical experience has been suggested with respect to the technique in changing dysfunctional beliefs. When the client is not emotionally immersed in the memory, the technique is ineffective in meeting clients’ needs.

## Limitations and Future Directions

The sample is unbalanced with regard to gender and refers to a specific population. In this sample, participants were highly educated and almost half of the sample was constituted by psychologists. This limits the generalization of our results since psychologists are more familiar with the concept of “basic emotional need” and are probably more aware than the general population of their frustrated needs. However, none of the participants had previous experience with ImR, nor with group ImR. Future studies should include a more heterogeneous sample.

Ultimately, the absence of long-term follow-up does not allow us to monitor whether changes in beliefs have remained stable over time.

## Data Availability Statement

The raw data supporting the conclusions of this article will be made available by the authors, without undue reservation.

## Ethics Statement

The studies involving human participants were reviewed and approved by the Guglielmo Marconi University Ethical Committee. The patients/participants provided their written informed consent to participate in this study.

## Author Contributions

All authors listed have made a substantial, direct, and intellectual contribution to the work, and approved it for publication.

## Conflict of Interest

The authors declare that the research was conducted in the absence of any commercial or financial relationships that could be construed as a potential conflict of interest.

## Publisher’s Note

All claims expressed in this article are solely those of the authors and do not necessarily represent those of their affiliated organizations, or those of the publisher, the editors and the reviewers. Any product that may be evaluated in this article, or claim that may be made by its manufacturer, is not guaranteed or endorsed by the publisher.

## Footnotes

1
https://www.questionpro.com
