# Supplementary material for: Group Imagery Rescripting on Childhood Memories Delivered via Telehealth: A Preliminary Study
Source: Front Psychiatry. 2022 Jun 23;13:862289. doi: 10.3389/fpsyt.2022.862289 (PMC9263974; doi:10.3389/fpsyt.2022.862289)
Supplement: Supplementary file 1 [file Data_Sheet_1.PDF]

IMAGERY INSTRUCTIONS

Shortly I am going to ask you to recall some memories of you as a child related to what you think about yourself today.
Sometimes the memories are not processed properly, especially if as a child you did not have someone who could help you understanding what was happening. The meaning we give to some experiences as a child can be very negative. For example, a child can conclude from an experience that he or she is bad, that others cannot be trusted, that it is dangerous to want the closeness of others, that one is to blame, should feel guilty and/or ashamed.
Even when we know from a rational perspective that such ideas are not correct, we continue to experience them as true.
We can't change what happened, and we can't erase those memories, but we can change the meaning we confer to those events.
We can try to change these meanings by talking and thinking, but we know from research that it is much more effective to use images.
We can have a much stronger impact on the brain when we imagine things than when we talk about them. Through imagination we will change perspective on what happened. We're not going to erase the memories, it is not possible, but we're going to help you to have a different view of what happened and experience a change in the meaning of those experiences.

The technique will trigger various feelings and new insights. Some of these may initially seem difficult to deal with, but experiencing strong emotions is a sign that the technique is effective.
During the Imagery if you find that a specific intervention is not enough for you this is not a problem at all, you can rewind the script and try something else.
We know it's better to use previous memories than more recent ones. Thus, if you have a choice, select a memory from when you were younger.
That was a pretty long explanation. Do you have any questions for now?

GROUP SAFE PLACE
Take a relaxed position and close your eyes (if you have trouble keeping your eyes closed, keep your eyes open and stare to a point on the floor)…
Bring to mind the image of a place that is safe for you, calm... you can be alone or with someone else, in a closed space or in an open space. It can be a realistic place or a place of fantasy. The important thing is that you visualize a place where you feel safe and quiet. Nod your head if you have visualized a place safe and quiet for yourself.

Describe the image you see with your mind's eye. What are the colors? What are the sounds? What are the smells? Try to feel what are the bodily sensations. Feel these feelings and try to take a snapshot of everything you see and feel right now. This will be your safe place.

Recent situation that activated the dysfunctional belief
Now I would like you to put aside the image of the safe place and to shift the attention to the last time you found yourself having that unpleasant thought that you wrote on the piece of paper.
When you have this recent situation in mind raise your hand.
Try to describe this scene in your mind as if it is happening right now.
Try to look at this scene with curiosity and focus on the images you see.

What colors do you see? Look at them.
Are there smells? Try to notice them.
Are there noises? Try to listen to them
Is there someone with you in the scene? What is he doing?
What are your thoughts?
What do you feel?
What emotions do you feel and
where you feel these emotions in your body?
Now I want you to get even closer to these emotions …

Floating Back
…and now I would like you to scroll through the memories of your past until you find an episode where you felt in the same way as a child. If more images reach the eye of your mind, choose the oldest memory. When you have the memory in mind, raise your hand.

1st Phase
Explore the memory that emerged taking the perspective of you as a child.
Try to describe in your mind this scene, it's happening right now. You are that child in that scene
Try to look, focus on the images you see.
What colors do you see? Look at them.
Are there smells? Try to notice them. Are there noises? Try to listen to them.
Is there someone with you in the scene? What is he doing?
What are your thoughts?
What do you feel? and where do you feel these emotions in your body?
Now I want you to get even closer to these emotions and think about what Your Child-self needs in this scene

2nd Phase
"Now I turn to you today, the adult person who is beyond the monitor"

Imagine to get in the scene of you as a child and to be the good parent you deserved, to comfort, protect and satisfy all needs of your Child-self. Get close to that little child and make him listen to you and talk to him with care and kindness.

Now tell to your Child-self: "I am here so that you are no longer uncomfortable. I will protect you. I will take care of you. You are precious and deserve protection."

You are the adult today, you are in that picture to respond to whatever you need as a kid.
Whatever your needs as a child, you are there to satisfy them completely. Now try to imagine what that child needs and try to fully meet his needs.

Turn to your Child-self and say: " You are little and like all children, you need care and you deserve your needs to be satisfied and I am going to give you everything you need. I know that others don't always understand you and realize that you have needs such as love, protection, or any other need. What I want you to know is that you deserve these things because all children deserve them and so do you.
Just because right now an important need is frustrated, it doesn't mean that you don't need it or that this need can't be satisfied.

Now I turn to you again, the Adult of today. Think about what yourself as a child needs and in the image make sure that he has everything he needs. Whatever he needs, you are there to give it to

him.

3rd Phase

Now I turn to you again as a child, the child of the image. "Have you seen your Adult-self taking care of your needs? try to think if there's anything else you need to feel completely satisfied,
If you can imagine what it would take to feel completely satisfied, imagine it happening right now.

Go on until all your needs are fully satisfied.
When you are completely satisfied, stay in this feeling of complete fulfillment and well-being. Try to locate where you feel it in your body, if you want you can put your hand where you feel this satisfaction. Enjoy this moment and this well-being.

Now, slowly, go back to the initial safe place. Take back that snapshot we took, you're there, you're completely safe.

Now I'm going to count to 10 and when you want, slowly, you can re-open your eyes
and slowly return to your breath.
